# The outcomes of margin status after sleeve lobectomy for patients of non–small cell lung cancer

**DOI:** 10.1111/1759-7714.14441

**Published:** 2022-05-05

**Authors:** Jianghao Ren, Mingyang Zhu, Yuanyuan Xu, Ruijun Liu, Ting Ren, Zhiyi Guo, Jiangbin Ren, Kan Wang, Qiang Tan

**Affiliations:** ^1^ Department of Thoracic Surgery Shanghai Chest Hospital, Shanghai Jiaotong University Shanghai China; ^2^ Huai'an First People's Hospital Nanjing Medical University Huai'an China; ^3^ The 4th Affiliated Hospital of Harbin Medical University Harbin China

**Keywords:** margin, sleeve lobectomy, surgery

## Abstract

**Background:**

Sleeve lobectomy is recognized as an alternative surgical operation to pneumonectomy because it preserves the most pulmonary function and has a considerable prognosis. In this study, we aimed to investigate the implications of residual status for patients after sleeve lobectomy.

**Methods:**

In this retrospective cohort study, we summarized 58 242 patients who underwent surgeries from 2015 to 2018 in Shanghai Chest Hospital and found 456 eligible patients meeting the criteria. The status of R2 was excluded. The outcomes were overall survival (OS) and recurrence‐free survival (RFS). We performed a subgroup analysis to further our investigation.

**Results:**

After the propensity score match, the baseline characteristic was balanced between two groups. The survival analysis showed no significant difference of overall survival and recurrence‐free survival between R0 and R1 groups (OS: *p* = 0.053; RFS: *p* = 0.14). In the multivariate Cox analysis, we found that the margin status was not a dependent risk factor to RFS (*p* = 0.119) and OS (*p* = 0.093). In the patients of R1, N stage and age were closely related to OS, but we did not find any significant risk variable in RFS for R1 status. In the subgroup analysis, R1 status may have a worse prognosis on patients with more lymph nodes examination. On further investigation, we demonstrated no differences among the four histological types of margin status.

**Conclusion:**

In our study, we confirmed that the margin status after sleeve lobectomies was not the risk factor to prognosis. However, patients with more lymph nodes resection should pay attention to the margin status.

## Graphical abstract

Sleeve lobectomy is recognized as an alternative surgical operation to pneumonectomy because it preserves the most pulmonary function and has a considerable prognosis. A positive margin is inevitable during the surgery sometimes. In our study, we confirmed that the margin status after sleeve lobectomies was not the risk factor to prognosis.

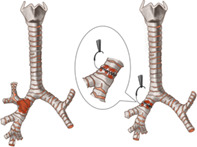

## INTRODUCTION

According to Global Cancer Statistics in 2020, there were nearly 19.3 million new cases and 10 million cancer deaths in 2020. Lung cancer is still at the top of lethal cancers.^1^ During the past few decades, pneumonectomy has remained an indispensable surgical operation for central localized non–small cell lung cancer (NSCLC) until the sleeve techniques were demonstrated to be superior. The sleeve lobectomy preserves the most pulmonary function and has a considerable prognosis. However, the decision to have a sleeve operation or the pneumonectomy depended on the surgeons individually under most circumstances and the relevant guidelines are not quite clear. A positive margin is inevitable during the surgery because of its central location. The International Association for the Study of Lung Cancer (IASLC) lung cancer staging project has extended the understanding of proposals for residual tumors for NSCLC in 2019.^2^ Although the adjuvant chemoradiotherapy will be taken to minimize the hazard of the positive margin, the influence of a positive margin on prognosis after the sleeve operation is still unclear. Should an extensive sleeve lobectomy, pneumonectomy, or no‐operation be performed after the frozen section report of a positive margin? It remains mysterious and needs further investigation. In our study, we compared the overall survival (OS) and recurrence‐free survival (RFS) between two margin statuses: R0 (negative) and R1 (positive residual under a microscope), which may contribute to the decision during the sleeve lobectomies.

## METHODS

### Patients

**FIGURE 1:**
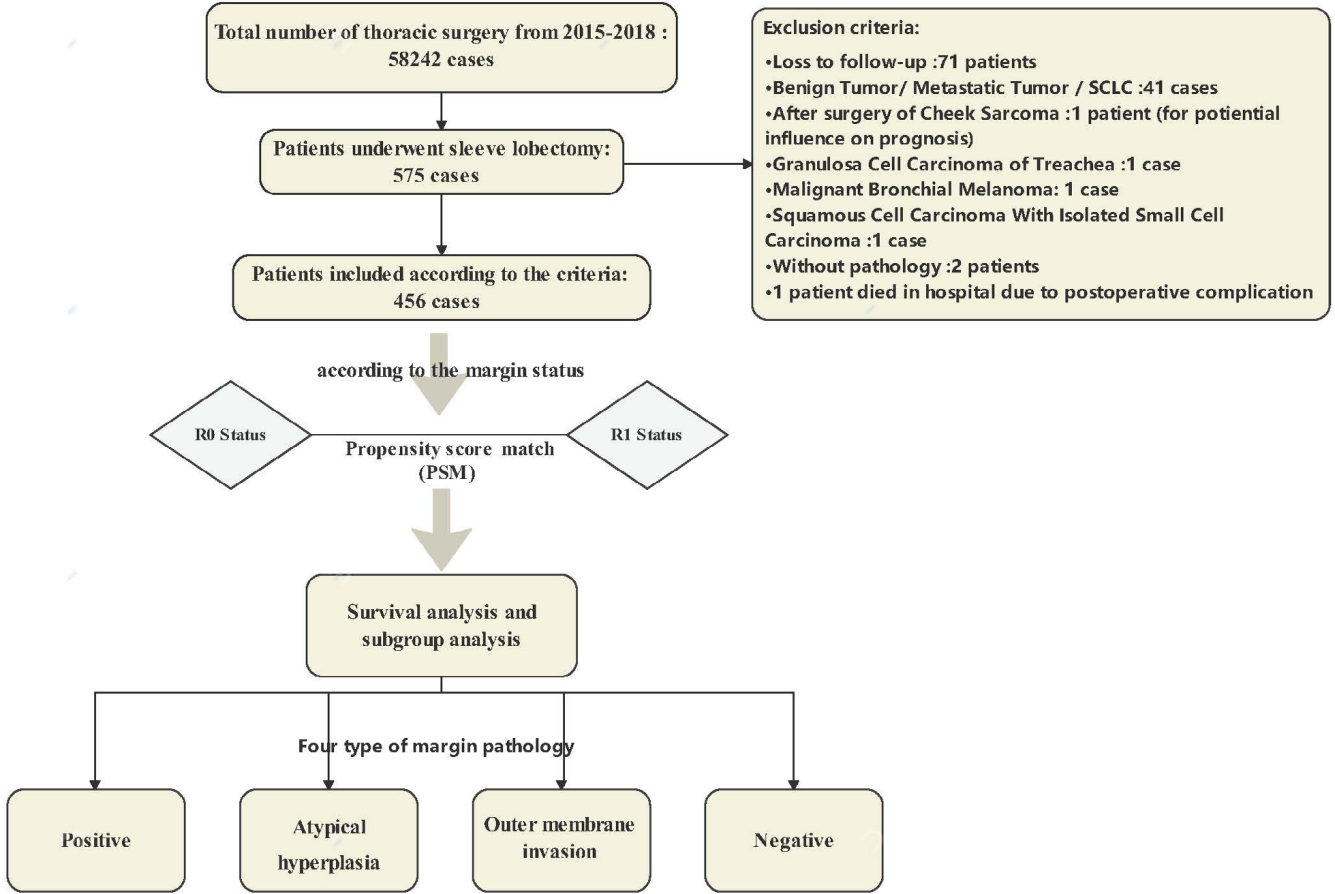
The design and participants of this study

In this study, we retrospectively collected 58 242 patients who underwent surgeries from 2015 to 2018 in Shanghai Chest Hospital. A total of 575 patients accepted sleeve operation. Only 456 cases met the criteria according to the inclusion principles. Among those people, 71 patients were lost to follow‐up, 20 were benign tumors, and 21 were metastatic lung tumors or small cell lung cancer. In addition, one case was excluded for postoperative cheek sarcoma, one for granulosa cell carcinoma of the trachea, one for malignant bronchial melanoma, one for squamous cell carcinoma with isolated small cell carcinoma, and two patients without pathology were also excluded (Figure 1).

### Study design

The clinical information of patients was retrieved from the clinical medical system in the Shanghai Chest Hospital. This study was a retrospective cohort study in a single center. We aimed to investigate the implication of margin status on the prognosis after sleeve lobectomy. According to residual status, we compared groups of two margin statuses: R0 (negative) and R1 (positive residual under the microscope). The status of R2 (macroscopic residual tumor) was not taken into consideration. We adopted the American Joint Committee on Cancer (AJCC) eighth edition to classify the TMN stage. The outcomes were OS and RFS. A subgroup for OS was performed to identify the patients at risk. Considering the pathology results of margin, we also compared the patients according to pathological type: positive, atypical hyperplasia/tumor in situ, outer membrane invasion, and negative.

### Statistical analysis

Continuous variables were expressed in the format of mean ± standard deviation (SD). Two independent sample *t*‐tests were applied to calculate the difference of the continuous variables and the categorical variables were analyzed by Fisher exact test or χ^2^ test. The baseline characteristic of the two groups was balanced by propensity scores match. The product‐limit method (Kaplan–Meier method) and the log‐rank test were used to evaluate and compare the OS and RFS. The univariate Cox regression analysis and multivariate Cox regression analysis where we used the method of “enter” were adopted to select the risk factors correlated to RFS/OS after sleeve lobectomies. A subgroup analysis of OS between R0 and R1 was carried out based on Cox analysis. We executed the pairwise comparisons using the log‐rank test for four types of histology and used the method of Benjamini‐Hochberg (BH) to adjust the *p*‐value. The *p*‐value of 0.05 in the study was deemed as a borderline of significant difference. The statistic procedure was assisted by software of SAS version 9.4 and R software version 4.0.3.

### Preoperative preparation and surgical techniques

All the patients before surgery had multidisciplinary consultation and comprehensive examinations. The enhanced thorax computed tomography (CT) scan was a conventional examination to have the preliminary recognition of the lesion. An enhanced CT or magnetic resonance imaging (MRI) of the head plus an optional positron emission tomography (PET)‐CT was used to evaluate the distant progress and mediastinal lymph nodes metastatic. A biopsy through the bronchoscope was necessary to specify the extent of the tumor and the histology whether it was small cell lung cancer. The surgical tolerability was assessed by pulmonary function, echocardiography, electrocardiograph, plate movement, and arterial blood gas analysis.

In our hospital, we mainly have three surgical techniques: open, video‐assisted thoracic surgery (VATS), and robot‐assisted thoracic surgery (RATS) from 2015 to 2018. The thoracoscope operation was mainly manipulated by a single aperture or three apertures. The patients with great vessels invasion, such as superior vena cava invasion and pulmonary artery, would accept angioplasty after thorough evaluation. The frozen section pathology was routinely performed to confirm the status of the margin. When it was R1 (positive under the microscope), the corresponding surgical decision depended on surgeons individually whether to extend resection of sleeve operation, pneumonectomy, or if no extra operation should be taken. As for lymph nodes examination, we routinely resected groups 2R, 4R, 7, 8, 9, 10R, and 11R for right‐side surgery and 4L, 5, 6, 7, 8, 9, 10L, and 11L for left‐sided surgery.

## RESULTS

### Baseline characteristics of R0 and R1 groups

The baseline characteristics of the two groups were listed in Table 1. The major histology in the sleeve operation was squamous cell carcinoma that tended to be located in the center. There was only one patient who died in hospital because of postoperative complications, and we excluded this case from the list. In our study, 10 patients underwent superior vena cava replacement and 56 patients suffered pulmonary artery angioplasty. A majority of patients had the T1–T2 stage, and it seemed that the N stage varied averagely from N0 to N2. In addition, approximately 13.60% patients accepted neoadjuvant therapy and 71.18% patients accepted adjuvant therapy.

**TABLE 1:** Baseline characteristics of patients suffering sleeve lobectomy before PSM

Variable	Summarize	R0 group	R1 group	*p* value
Sex (*n*)				
Male	416	333 (91.74%)	83 (89.25%)	0.4183
Female	40	30 (8.26%)	10 (10.75%)	
Age (y)	60.46 ± 8.93	60.99 ± 8.88	58.40 ± 8.88	0.0123 < 0.05
BMI (kg/m^2^)	23.23 ± 2.97	23.28 ± 2.91	23.06 ± 3.22	0.5378
Hospital days (d)	16.15 ± 8.13	17.77 ± 7.41	17.63 ± 10.36	0.1063
Death in hospital (*n*)	1	1	0	
Laterality (*n*)				0.0040 < 0.05
Left	285	215 (59.23%)	70 (70.27%)	
Right	171	148 (40.77%)	23 (24.73%)	
Tumor size (cm)	3.77 ± 1.53	3.78 ± 1.54	3.72 ± 1.49	0.7414
Surgical technology (*n*)				0.5196
Open	387	310 (85.40%)	77 (82.80%)	
VATS + RATS	69	53 (14.60%)	16 (17.20%)	
T stage (*n*)				0.1367
T1 + T2	332	270 (74.38%)	62 (66.67%)	
T3	56	45 (12.40%)	11 (11.83%)	
T4	68	48 (13.22%)	20 (21.51%)	
N stage (*n*)				0.0037 < 0.05
N0	167	146 (40.22%)	21 (22.58%)	
N1	159	116 (31.96%)	43 (46.24%)	
N2	130	101 (27.82%)	29 (31.18%)	
Pathological stage (*n*)				0.0143 < 0.05
I	107	94 (25.10%)	13 (13.98%)	
II	157	127 (34.99%)	30 (32.26%)	
III	192	142 (39.13%)	50 (53.76%)	
Lymph nodes resection (*n*)				
Total	16.29 ± 6.58	16.31 ± 6.80	16.20 ± 5.70	0.8771
N1	6.67 ± 3.66	6.62 ± 3.66	6.85 ± 3.68	0.5943
N2	9.60 ± 4.94	9.66 ± 5.11	9.37 ± 4.27	0.5648
Histology (*n*)				0.1671
SCC	340	274 (75.48%)	66 (70.97%)	
Adenocarcinoma	63	52 (14.33%)	11 (11.83%)	
ACC and others	53	37 (10.19%)	16 (17.20%)	
Superior vena cava invasion				0.1256
No	446	357 (98.35%)	89 (95.70%)	
Yes	10	6 (1.65%)	4 (4.30%)	
Pulmonary artery angioplasty				0.1555
No	400	314 (86.50%)	86 (92.47%)	
Yes	56	49 (13.50%)	7 (7.53%)	
Neoadjuvant therapy (*n*)				0.0621
No	394	308 (84.85%)	86 (92.47%)	
Yes	62	55 (15.15%)	7 (7.53%)	
Adjuvant therapy (*n*)				0.6026
No	125	102 (28.10%)	23 (24.73%)	
Yes	331	261 (71.90%)	70 (75.27%)	
Comorbidity (*n*)				
Cardiovascular system	181	141	40	
Nervous system	240	192	48	
Hypertension	87	69	18	
Diabetes	42	35	7	
FEV1	2.88 ± 0.38	2.89 ± 0.39	2.84 ± 0.37	0.3183
FEV1%	79.63 ± 15.76	79.97 ± 15.90	78.04 ± 15.03	0.32
DLCO%	86.90 ± 19.59	87.37 ± 19.81	84.70 ± 18.46	0.2692

### Propensity scores match to balance the baseline

**FIGURE 2:**
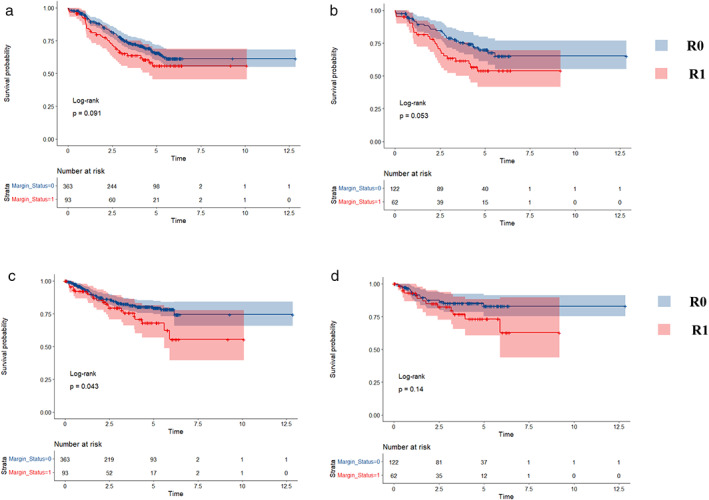
Survival analyses between R0 and R1 before PSM and after PSM. (a) Overall survival before PSM. (b) Overall survival after PSM. (c) Recurrence‐free survival before PSM. (d) Recurrence‐free survival after PSM

The significant differences between two groups were observed in age, laterality, N stage and clinical stage before PSM. The ratio of PSM was 1:2 and the caliper was set at 0.02. The results of PSM were recorded in Table 3. Before the balance, the comparison of OS between two groups through log‐rank test showed that the *p* value was 0.091. After PSM, a difference that nearly reached statistical significance (*p* = 0.053) between R0 and R1 groups was observed. As for RFS, the *p* values were 0.043 (before PSM) and 0.14 (after PSM), which showed no obvious statistical differences (Figure 2).

### Postoperative complications of patients during hospitalization

The most frequent complications in the R0 group were hypokalemia (*n* = 54), atelectasis (*n* = 63), and hypoxemia (*n* = 45, three patients had ARDS). The second was bacterial infection or pneumonia, and one patient was infected by MASA. In the R1 group, hypoxemia (*n* = 21) and hypokalemia (*n* = 23) were also the common postoperative complications. In this group, one patient with empyema was infected by MASA and another one suffered acute syndrome derived from anastomotic stenosis. Seven patients had anastomotic fistula in the R0 group and three in the R1 group, respectively. Cases of BPF were found in four patients and one patient in two groups. Only one patient had a re‐operation because of an anastomotic fistula (Table 2).

**TABLE 2:** Postoperative complications during hospitalization of patients after sleeve pneumonectomy

Diseases	Total cases	R0 group (*n* = 363)	R1 group (*n* = 93)
Hypercapnia	19	6	13
Hypoxemia	66	45 (3 for ARDS)	21
Acid‐base disturbance	153	110	40
Hypokalemia	77	54	23
Bacterial infection or pneumonia	39	38 (1 for MASA)	11
Atelectasis or pneumothorax	77	63	14
Anastomotic fistula	10	7 (1 for re‐operation)	3
Anastomotic‐stenosis	4	2	2 (1 for acute syndrome)
Empyema	10	7	3 (1 for MASA)
BPF	5	4	1
Respiratory failure	3	2	1
Pulmonary edema	1	1	0
Hemoptysis	2	1	1
Chylothorax	2	0	2
Embolization	9	7	4
ACS	3	2	1
Heart hernia	2	1	1
Arrhythmia	6	4	2

**TABLE 3:** Baseline characteristics of patients suffering sleeve lobectomy after PSM

Variable	Summarize	R0 group	R1 group	*p* value
Sex (*n*)				0.617
Male	164	110 ( 90.2%)	54 (87.1%)	
Female	20	12 (9.8%)	8 (12.9%)	
Age (y)	60.41 ± 8.254	60.93 ± 8.11	59.37 ± 8.51	0.226
BMI (kg/m^2^)	23.40 ± 3.02	23.60 ± 2.98	23.02 ± 3.08	0.219
Hospital days (d)	15.89 ± 5.86	15.66 ± 6.36	16.35 ± 4.729	0.446
Laterality (*n*)				0.855
Left	126	83 (68.0%)	43 (69.4%)	
Right	58	39 (320%)	19 (30.6%)	
Tumor size (cm)	3.75 ± 1.56	3.73 ± 1.56	3.80 ± 1.57	0.763
Surgical technology (*n*)				0.527
Open	154	104 (85.2%)	50 (80.6%)	
VATS + RATS	30	18 (14.8%)	12 (19.4%)	
T stage (*n*)				0.155
T1 + T2	146	100 (82/0%)	46 (74.2%)	
T3	17	12 (9.8%)	5 (8.1%)	
T4	21	10 (8.2%)	11 (17.7%)	
N stage (*n*)				0.641
N0	51	35 (28.7%)	16 (25.8%)	
N1	76	52 (42.6%)	24 (38.7%)	
N2	57	35 (28.7%)	22 (35.5%)	
Pathological stage (*n*)				0.468
I	39	28 (23.0%)	11 (17.7%)	
II	70	48 (39.3%)	22 (35.5%)	
III	75	46 (37.7%)	29 (46.8%)	
Lymph nodes resection (*n*)				
Total	16.20 ± 6.11	16.48 ± 6.28	15.65 ± 5.763	0.38
N1	6.96 ± 3.51	7.27 ± 3.52	6.34 ± 3.45	0.089
N2	9.24 ± 4.47	9.20 ± 4.48	9.31 ± 4.48	0.885
Histology (*n*)				0.954
SCC	137	91 (74.6%)	46 (74.2%)	
Non‐SCC	47	31 (25.4%)	16 (25.8%)	
Superior vena cava invasion				0.112
No	180	121 (99.2%)	59 (95.2%)	
Yes	4	1 (0.8%)	3 (4.8%)	
Pulmonary artery angioplasty				0.793
No	167	110 (90.2%)	57 (91.9%)	
Yes	17	12 (9.8%)	5 (8.1%)	
Neoadjuvant therapy (*n*)				
No	167	111 (91.0%)	56 (90.3%)	
Yes	17	11 (9.0%)	6 (9.7%)	
Adjuvant therapy (*n*)				0.725
No	48	33 (27.0%)	15 (24.2%)	
Yes	136	89 (73.0%)	47 (75.8%)	

**TABLE 4:** Univariate and multivariate analyses of prognostic factors of OS and RFS

Variable	RFS		OS	
	Univariate analyses	Multivariate analyses	Univariate analyses	Multivariate analyses
	HR (95% CI) *p* value	HR (95% CI) *p* value	HR (95% CI) *p* value	HR (95% CI) *p* value
Age at diagnosis	1.032 (1.003–1.060) 0.027	1.037 (1.008–1.067) 0.011	1.032 (1.011–1.054) 0.003	1.033 (1.011–1.054) 0.003
Sex	1.143 (0.550–2.375) 0.720		0.700 (0.356–1.376) 0.301	
BMI	0.976 (0.906–1.052) 0.523		0.970 (0.916–1.028) 0.304	
In‐hospital days	1.021 (1.001–1.040) 0.034	1.013 (0.991–1.036) 0.260	1.022 (1.008–1.037) 0.002	1.013 (0.999–1.028) 0.075
Laterality (left/right)	0.748 (0.465–1.202) 0.230		0.758 (0.530–1.086) 0.131	
Neoadjuvant therapy	1.366 (0.753–2.476) 0.304		1.780 (1.168–2.713) 0.007	1.704 (1.106–2.624) 0.016
Adjuvant therapy	0.936 (0.568–1.542) 0.795		0.901 (0.620–1.310) 0.584	
Carina reconstruction	2.109 (1.014–4.385) 0.046	1.844 (0.772–4.401) 0.168	1.266 (0.644–2.491) 0.494	
Superior vena cava invasion	1.021 (0.142–7.359) 0.984		4.440 (1.947–10.13) 0.000	2.667 (1.144–6.215) 0.023
Surgical technique				
Open	Control in dummy variable			
VATS + RATS	0.899 (0.508–1.592) 0.716		Reference 0.951 (0.579–1.561) 0.842	
Pulmonary artery angioplasty	1.072 (0.515–2.229) 0.853		1.398 (0.841–2.325) 0.196	
Margin status (positive/negative)	0.598 (0.368–0.972) 0.038	1.506 (0.900–2.519) 0.119	1.390 (0.947–2.041) 0.093	
Tumor size	1.186 (1.022–1.375) 0.024	1.189 (0.999–1.415) 0.051	1.267 (1.136–1.413) 0.000	1.228 (1.077–1.401) 0.002
Histology	0.668		0.132	
SCC	Control in dummy variable		Reference	
Adenocarcinoma	0.810 (0.402–1.629) 0.554		1.474 (0.950–2.288) 0.083	
ACC and others	0.745 (0.341–1.629) 0.745		0.792 (0.435–1.441) 0.445	
T stage	0.314		0.173	
T1 + T2	Control in dummy variable		Reference	
T3	1.091 (0.539–2.208) 0.808		1.558 (0.971–2.499) 0.066	
T4	1.577 (0.877–2.838) 0.128		1.180 (0.713–1.952) 0.519	
N stage	0.015	0.072	0.000	0.000
N0	Control in dummy variable	Reference	Reference	Reference
N1	1.309 (0.750–2.284) 0.343	1.574 (0.759–3.264) 0.223	1.747 (1.069–2.856) 1.747	1.787 (0.946–3.376) 0.073
N2	2.198 (1.270–3.805) 0.005	3.016 (1.169–7.784) 0.022	4.705 (2.996–7.389) 0.000	5.226 (2.265–12.05) 0.000
No. of N1 resection	0.952 (0.892–1.016) 0.141		1.020 (0.976–1.066) 0.382	
No. of N2 resection	0.950 (0.904–0.999) 0.045	0.999 (0.913–1.093) 0.982	0.987 (0.953–1.022) 0.458	
No. of total nodes	0.956 (0.921–0.993) 0.020	0.953 (0.891–1.019) 0.158	1.001 (0.975–1.026) 0.969	
Pathological stages	0.028	0.659	0.000	0.815
Stage I	Control in dummy variable	Reference	Reference	Reference
Stage II	2.113 (0.881–5.068) 0.094	0.738 (0.317–1.718) 0.481	1.753 (0.977–3.145) 0.060	0.933 (0.438–1.986) 0.857
Stage III	3.412 (1.494–7.792) 0.003	0.603 (0.202–1.799) 0.365	4.176 (2.442–7.142) 0.000	0.758 (0.284–2.023) 0.581

### Risk factors correlated to prognosis after sleeve lobectomy

The variables with *p*‐value <0.05 were selected into the multivariable Cox model and the method was “enter.” In Table 4, we found that age at diagnosis (95% CI, 1.008–1.067, *p* = 0.011) and N stage especially N2 stage (95% CI, 1.169–7.784, *p* = 0.022) would influence the post‐operative recurrence, but there was a borderline effect in which survival analysis demonstrated a somewhat higher hazard rate for tumor size (95% CI, 0.999–1.415, *p* = 0.051). In the analysis of overall survival, age at diagnosis (95% CI, 1.011–1.054, *p* = 0.003), neoadjuvant therapy (95% CI, 1.106–2.614, *p* = 0.016), superior vena cava invasion (95% CI, 1.144–6.215, *p* = 0.023), tumor size (95% CI, 1.077–1.401, *p* = 0.002), and N stage (95% CI, 5.265–12.05, *p* = 0.000) had significant differences. In addition, we also performed a survival analysis based on the Cox regression for the R1 population. Age at diagnosis (95% CI, 1.014–1.117, *p* = 0.012) and N stage (95% CI, 1.205–35.52, *p* = 0.030) was still confirmed to be significant to OS, but to our surprise, we did not find any risk factors to RFS. In addition, the margin status was not a relevant factor to RFS or OS.

### Subgroup analysis and pairwise comparisons between histology types

**FIGURE 3:**
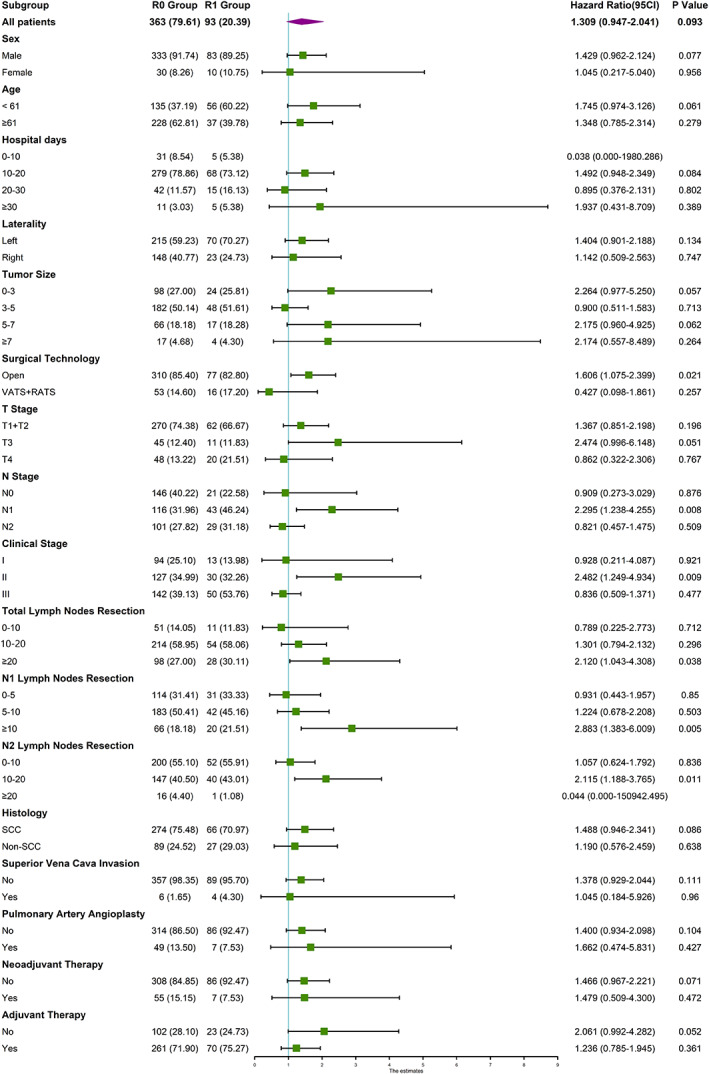
Subgroup analysis of margin status for overall survival

Because the survival analysis using the log‐rank test for OS showed a borderline statistical significance (*p* = 0.053), we conducted a subgroup analysis for OS for further study (Figure 3). In the subgroup analysis for OS, we discovered that patients with open techniques, N1 stage, clinical stage II may harm prognosis. Besides, it seemed that more lymph nodes examination would cause poorer survival when the margin was positive.

**FIGURE 4:**
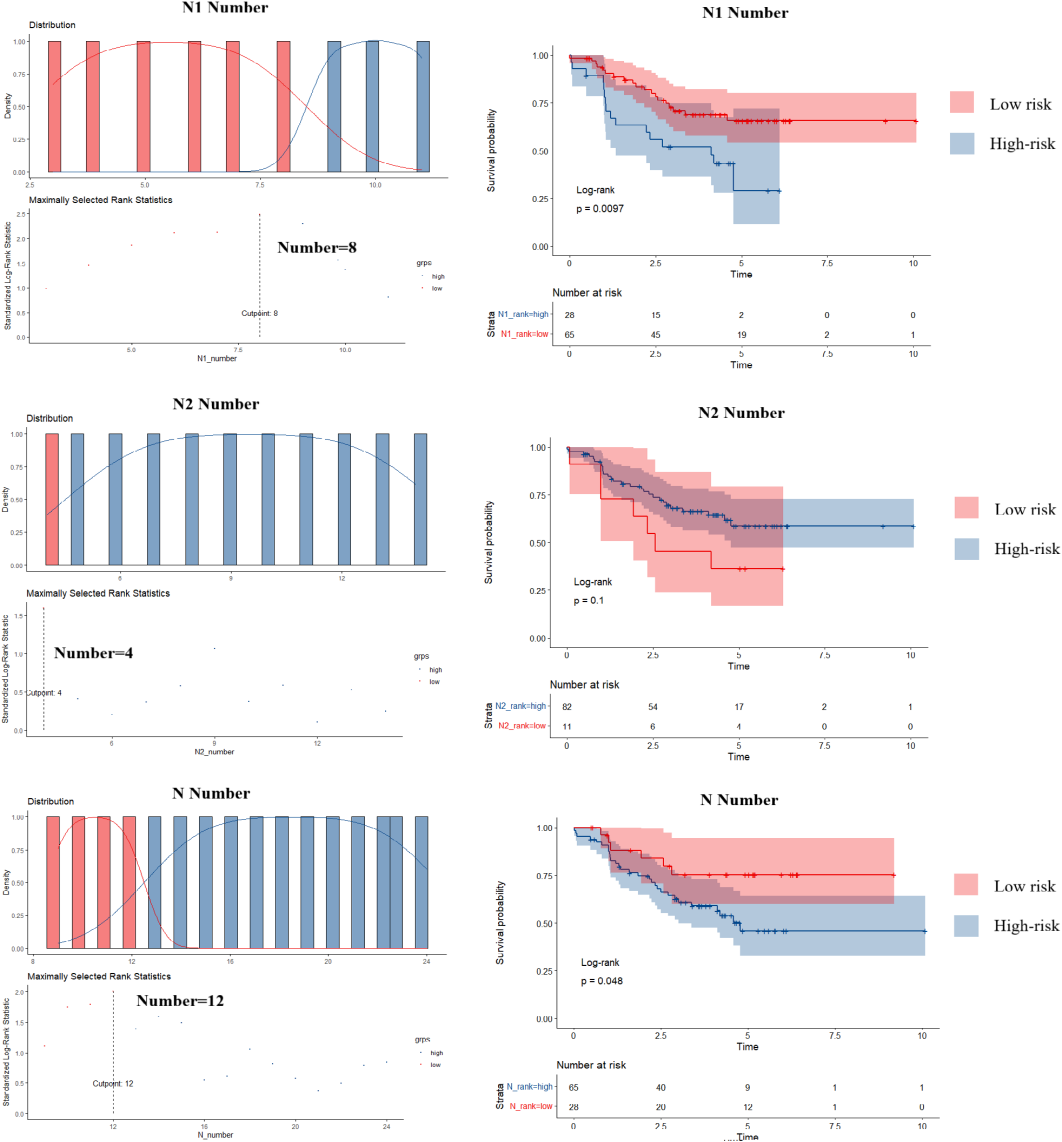
The cutoff of the lymph nodes resection (the cutoffs were 8, 4, and 12 for N1, N2, and N number)

Furthermore, we aimed at the number of lymph nodes resection and had a cutoff analysis based on KM curve and the log‐rank test. The breakpoints of N number and N1 number were 12 and 8, respectively. As for the N2 number, although the Figure showed the breakpoint was 4, the log‐rank test and survival curve concluded a *p*‐value of no statistical implication (*p* = 0.1) (Figure 4).

**FIGURE 5:**
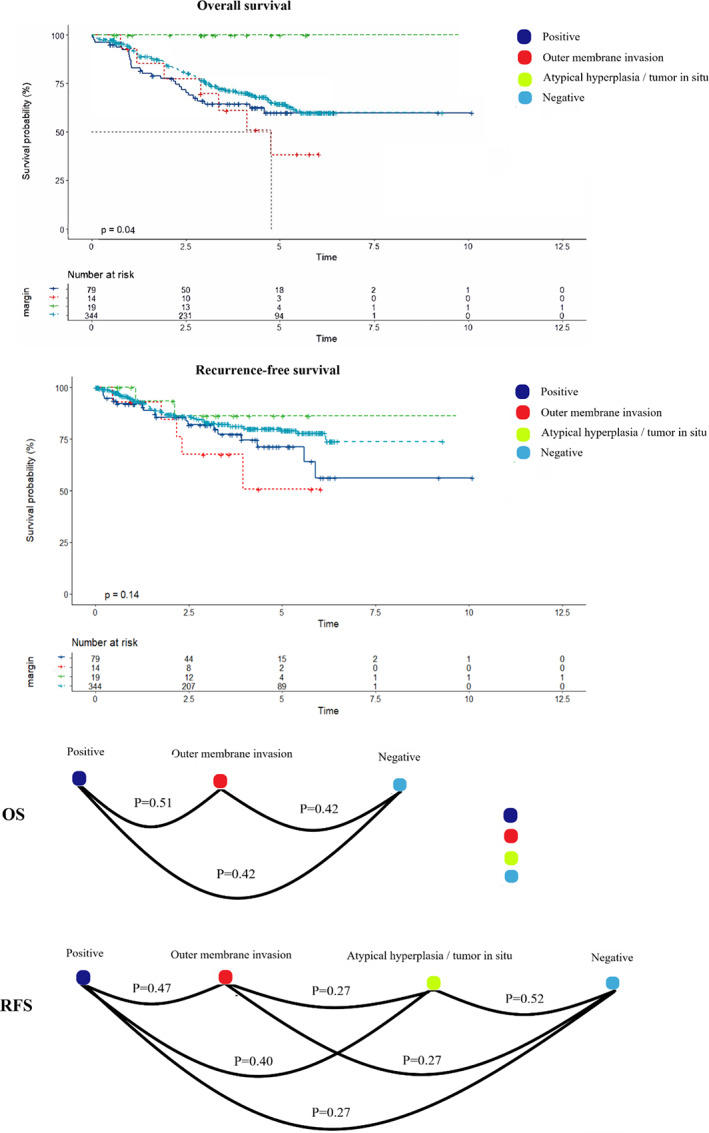
Pairwise comparisons of histological types of margin status

There were no patients with the margin of atypical hyperplasia or tumor in situ who died after surgeries, and we compared the other three histology types, which indicated no significant differences. The pairwise comparisons using the log‐rank test for RFS revealed no differences (Figure 5).

## DISCUSSION

With the development of surgical techniques, sleeve operation has been widely recognized as an alternative to pneumonectomy when it is a centrally located lesion. Sleeve lobectomy not only preserves maximum lung function (PF), but also has a better prognosis than pneumonectomy. However, the sleeve operations are always companied with a disputable problem that the margin of resection will be inevitably positive sometimes. In response to this, Lee et al.^3^ explored a novel evaluation for resectability of sleeve lobectomy with the aid of CT features. Some articles reported the frequency of R1 resection ranging from 1.2% to 17%.^4^, ^5^ The management of a positive margin is always controversial and usually depends on the decision of individual surgeons. There has not been any clear consensus to guide the surgeon on whether to have extensive sleeve lobectomy, a pneumonectomy, or no‐operation after reporting the frozen section results.

In our study, we found that the margin status was not related to the prognosis of both RFS and OS although the OS between R0 and R1 tended to be significant (*p* = 0.053). It indicated that a positive margin might not be that serious and an extensive sleeve or a pneumonectomy might not necessary. Similarly, Hong et al.^6^ drew the homologous conclusion in 2020 that R1 after sleeve operations generally showed long‐term survival and are not significantly jeopardized in terms of oncologic outcomes. Among those participants, nearly 75.8% (47/62) patients accepted adjuvant therapy including radiology. The local control of disease may be in part because of radiotherapy that was proposed by Massard et al.^7^ as early as 2000. As National Comprehensive Cancer Network clinical practice guidelines in oncology (NCCN guidelines) refers, it is widely recognized that radiotherapy plus selective chemotherapy is the standard treatment for positive margin. However, the radiotherapy sometimes increases the anastomotic complications to some extent, especially in advanced patients. The IASLC lung cancer staging project argued that the R status has some importance in prognosis evaluation and should be considered in the design of trials.^2^ Because Wind et al.^4^ interpreted that the negative effect of R1status has mainly been observed in patients with clinical stage III–IV and the patients of R0 with stage I–II had obvious better prognosis compared to R1 patients. However, in our subgroup and multivariable analysis, we did not find such above tendency and the clinical stage was even not an independent risk factor to both OS and RFS.

In the past relative study, the 5‐year OS of patients with R0 resection who accepted sleeve lobectomy varied from 30% to 60%.^8^, ^9^ In our study, the 5‐year OS and RFS of R0 resection had reached 64.9% and 78.3%, respectively, and reached 55% and 62.4% for R1 resection, which was closed to R0 resection. Considering the potential implication of margin histology, we further discussed its histologic subgroup. Nevertheless, there were no patients with atypical hyperplasia or tumor in situ who died and no statistical difference of OS was found between the other three types. Neither, the RFS between four types mentioned above had no significant difference. However, some articles mentioned that the margin of in situ did not have any negative influence on survival and had comparable progress to radical resection, and they also insisted that the invasive margin had a progressive tendency of recurrence.^6^, ^7^ Therefore, our study needs further investigation to analyze the histology subgroup.

Compared to margin status, the N stage was a more momentous risk factor. Because the lymph system was the potential metastatic pathway of tumor cells, and the positive mediastinal lymph nodes indicated to be prone to local progress. The 5‐year OS of N0, N1, and N2 were 78.5%, 66.0%, 37.4%, and the corresponding RFS were 80.9%, 76.2%, 65.3% in our study where significant differences were found among them. In the subgroup analysis, R1 status had a potentially negative effect on patients with more lymph nodes resection. Lymph node dissection helped remove potential local micro‐metastases, however, it disrupts the patient's innate immune system. How to maintain a balance between them needs to be determined. In the subgroup analysis, we also found that the margin status might affect the prognosis of patients with clinical II or N1 stage. We conjectured that, in patients of N2 stage or clinical stage III‐IV, the disease itself had a more important contribution to tumor progress compared to margin status. However, we cannot explain why margin status was not a risk factor to patients with early‐stage such as clinical stage I and N0 stage.

In addition to these factors, neoadjuvant chemotherapy is also an emerging technique to sleeve lobectomy. In the multivariate analysis, we concluded that neoadjuvant chemotherapy could not improve the outcome of RFS, but was an independent variable to OS. As early as 1997, Rendina et al.^10^ had demonstrated the safety and efficacy of bronchus reconstruction after induction chemotherapy for NSCLC. A series of trials have been currently undertaken. It has been recognized that patients with neoadjuvant chemotherapy can acquire a better prognosis^11^, ^12^ and the neoadjuvant chemotherapy will not increase surgical morbidity, anastomotic complications as well as mortality.^13^, ^14^ However, neoadjuvant radiology is associated with increased anastomotic complications such as anastomotic fistula.^15^

Our study has some limitations. First, it was a retrospective cohort study in a single center of Shanghai Chest Hospital rather than a randomized controlled trial although we performed the propensity scores to balance the baseline. Second, the sample size of our study was a little small, which took some bias in the survival analysis. Third, the follow‐up time of 5 years was insufficient and not all patients were followed up for 5 years.

## CONFLICT OF INTEREST

The authors declare no conflicts of interest.

## References

[1] Sung H , Ferlay J , Siegel RL , Laversanne M , Soerjomataram I , Jemal A , et al. Global cancer statistics 2020: GLOBOCAN estimates of incidence and mortality worldwide for 36 cancers in 185 countries. CA Cancer J Clin. 2021;71:209–49. 33538338 10.3322/caac.21660

[2] Edwards JG , Chansky K , Van Schil P , et al. The IASLC lung cancer staging project: analysis of resection margin status and proposals for residual tumor descriptors for non‐small cell lung cancer. J Thorac Oncol. 2020;15:344–59. 31731014 10.1016/j.jtho.2019.10.019

[3] Lee JH , Yoon SH , Kim YT , Kang CH , Park IK , Park S , et al. Sleeve lobectomy for non‐small cell lung cancers: predictive CT features for resectability and outcome analysis. AJR Am J Roentgenol. 2019;213:807–16. 31166766 10.2214/AJR.19.21258

[4] Wind J , Smit EJ , Senan S , Eerenberg JP . Residual disease at the bronchial stump after curative resection for lung cancer. Eur J Cardiothorac Surg. 2007;32:29–34. 17466532 10.1016/j.ejcts.2007.04.003

[5] Pezzetta E , Stupp R , Zouhair A , et al. Comparison of neoadjuvant cisplatin‐based chemotherapy versus radiochemotherapy followed by resection for stage III (N2) NSCLC. Eur J Cardiothorac Surg. 2005;27:1092–8. 15896624 10.1016/j.ejcts.2005.02.035

[6] Hong TH , Kim J , Shin S , et al. Clinical outcomes of microscopic residual disease after bronchial sleeve resection for non‐small cell lung cancer. J Thorac Cardiovasc Surg. 2021;161:267–77. 10.1016/j.jtcvs.2020.02.079 32249083

[7] Massard G , Doddoli C , Gasser B , Ducrocq X , Kessler R , Schumacher C , et al. Prognostic implications of a positive bronchial resection margin. Eur J Cardiothorac Surg. 2000;17:557–65. 10814919 10.1016/s1010-7940(00)00384-5

[8] Cerfolio RJ , Bryant AS : Surgical techniques and results for partial or circumferential sleeve resection of the pulmonary artery for patients with non‐small cell lung cancer. Ann Thorac Surg 83:1971–6; discussion 1976–7, 2007 17532380 10.1016/j.athoracsur.2007.01.048

[9] Kim HK , Cho JH , Choi YS , Zo JI , Shim YM , Park K , et al. Outcomes of neoadjuvant concurrent chemoradiotherapy followed by surgery for non‐small‐cell lung cancer with N2 disease. Lung Cancer. 2016;96:56–62. 27133751 10.1016/j.lungcan.2016.03.016

[10] Rendina EA , Venuta F , De Giacomo T , et al. Safety and efficacy of bronchovascular reconstruction after induction chemotherapy for lung cancer. J Thorac Cardiovasc Surg. 1997;114:830–5; discussion 835‐7. 9375614 10.1016/S0022-5223(97)70088-6

[11] Chen Y , Zhang L , Yan B , Zeng Z , Hui Z , Zhang R , et al. Feasibility of sleeve lobectomy after neo‐adjuvant chemo‐immunotherapy in non‐small cell lung cancer. Transl Lung Cancer Res. 2020;9:761–7. 32676337 10.21037/tlcr-20-539 PMC7354158

[12] Gómez‐Caro A , Boada M , Reguart N , et al. Sleeve lobectomy after induction chemoradiotherapy. Eur J Cardiothorac Surg. 2012;41:1052–8. 22223693 10.1093/ejcts/ezr184

[13] Comacchio GM , Schiavon M , Azzolina D , Mammana M , Marulli G , Zuin A , et al. Does induction therapy increase anastomotic complications in bronchial sleeve resections? World J Surg. 2019;43:1385–92. 30659342 10.1007/s00268-019-04908-0

[14] Milman S , Kim AW , Warren WH , et al: The incidence of perioperative anastomotic complications after sleeve lobectomy is not increased after neoadjuvant chemoradiotherapy. Ann Thorac Surg 88:945–50; discussion 950–1, 2009 19699925 10.1016/j.athoracsur.2009.05.084

[15] Koryllos A , Lopez‐Pastorini A , Zalepugas D , Ludwig C , Hammer‐Helmig M , Stoelben E . Bronchus anastomosis healing depending on type of neoadjuvant therapy. Ann Thorac Surg. 2020;109:879–86. 31843636 10.1016/j.athoracsur.2019.10.049

